# Learning Curve in Left Ventricular Assist Device Implantation: Low Volumes Do Not Equate Bad Outcomes

**DOI:** 10.21470/1678-9741-2021-0498

**Published:** 2022

**Authors:** Mélanie Hébert, Pierre-Emmanuel Noly, Yoan Lamarche, Olina Dagher, Ismail Bouhout, Elie Hage-Moussa, Thierry Lévesque, Geneviève Giraldeau, Normand Racine, Anique Ducharme, Michel Carrier

**Affiliations:** 1 Department of Cardiac Surgery, Montreal Heart Institute, Montreal, Canada; 2 Department of Cardiology, Montreal Heart Institute, Montreal, Canada

**Keywords:** Circulatory Support Devices (LVAD, RVAD, BVAD, TAH), Heart Failure (Incl Diagnosis, Assessment, Treatment), Outcomes (Incl Mortality, Morbidity, Survival, Etc.), Postoperative Care

## Abstract

**Introduction:**

Most implantations of left ventricular assist devices (LVAD) are performed in low-volume centers. This study aimed to evaluate the procedural learning curve of HeartMate II (HM2) implantations by comparing outcomes between two time periods in a low-volume center.

**Methods:**

All 51 consecutive patients undergoing HM2 implantation between January 2009 and December 2017 were reviewed and allocated into 2 groups: early-era group (from 2009 to 2014; n=25) and late-era group (from 2015 to 2017; n=26). The primary outcome was the 90-day mortality rate, and the secondary outcome was a composite of mortality, neurological event, reoperation for bleeding, need for temporary right ventricular assist device, and pump thrombosis at 90 days. Median follow-up time was 51 months (0-136). A cumulative sum (CUSUM) control analysis was used to establish a threshold of implantations that optimizes outcomes.

**Results:**

Patients in the early era had a higher rate of diabetes, previous stroke, and inotrope support before HM2 implantation. The 90-day mortality rate was not significantly higher in the early era (24% vs. 15%, P=0.43), but the composite endpoint was significantly higher (76% vs. 42%, P=0.01). The CUSUM analysis found a threshold of 23 operations after which the composite endpoint was optimized.

**Conclusion:**

Patients undergoing HM2 implantation in a low-volume center have improving outcomes with number of cases and optimized results after a threshold of 23 cases. Significant changes in patient selection, surgical techniques, and patient management might lead to improved outcomes after LVAD implantation.

**Table:** 

Abbreviations, Acronyms & Symbols				
BTT	= Bridge to transplantation		HM2	= HeartMate II
BVAD	= Biventricular assist device		HT	= Heart transplantation
CF-LVAD	= Continuous-flow left ventricular assist device		LOS	= Length of stay
CPB	= Cardiopulmonary bypass		LVAD	= Left ventricular assist device
CUSUM	= Cumulative sum		RVAD	= Right ventricular assist device
DT	= Destination therapy		SD	= Standard deviation
ICU	= Intensive care unit		TAH	= Total artificial heart
INTERMACS	= Interagency Registry for Mechanically Assisted Circulatory Support			

## INTRODUCTION

The use of continuous-flow left ventricular assist devices (CF-LVADs) in the management of patients with end-stage heart failure has been increasing over the years^[1,2]^. Many patients are either ineligible to heart transplantation (HT) or unable to wait for a graft due to a declining condition. CF-LVAD therapy is now recommended for selected patients with end-stage heart failure, either as a bridge to transplantation (BTT), bridge to candidacy, or destination therapy (DT)^[3]^.

Several studies have shown that outcomes after CF-LVAD implantation are related to annual center volume^[4-7]^. For this reason, current coverage by the Centers for Medicare & Medicaid Services requires a surgeon with at least 10 CF-LVAD implantations in the past three years^[8]^. Optimal volume thresholds remain controversial, but some authors suggest an optimal volume ranging between 20 and 50 annual implantations per center^[4,6]^. Better outcomes reported in high-volume centers may be related to patient selection, established processes for surgical management, postoperative care, and long-term medical follow-up^[5]^. Others have shown that the advantage of high-volume centers should be accounted for by surgeon volume^[9]^. The necessity of volume thresholds has also been disputed in favor of evaluating centers by demonstrated outcomes^[10]^.

In Canada, there is no true recommendation regarding volume thresholds for CF-LVAD implantation. In Quebec, the Institut National d’Excellence en Santé et en Services Sociaux requires a minimum of 10 implantations of CF-LVAD every three years to provide certification for a center. During the last eight years, <30 patients per year got a CF-LVAD implantation in the province of Quebec among three cardiac centers, the number of CF-LVAD patients varying from 0 to 11 depending on the center and year^[11]^. It is important to understand outcomes in low-volume centers as 43% of patients in the United States receive a CF-LVAD implantation in a center with <30 implantations per year^[4]^.

Given the prevalence of low-volume centers, studying outcomes in the face of this contingency is important to understand what can be improved with increasing number of cases. This study therefore aims to evaluate outcomes of HeartMate II™ (HM2; Abbott, Chicago, IL) in a low-volume center by investigating the impact of the learning curve on implantations.

## METHODS

### Study Population and Data Collection

This retrospective observational study was approved by the Institutional Review Board. All 51 consecutive patients who underwent HM2 implantation at the institution between January 2009 and December 2017 were reviewed. Preoperative characteristics, including demographics, medical history, clinical status at admission, biological parameters, echocardiography, and right heart catheterization were studied. Perioperative and postoperative data, including operative durations, concomitant procedures, postoperative complications, hospital length of stay (LOS), intensive care unit (ICU) LOS, and causes of death were also examined. Data were collected from electronic medical records, patients’ hospital charts, and clinical follow-up. Data collection ended in September 2020. Median follow-up was 51 months (0-136 months) and was 100% complete.

Patients were allocated into two groups based on timing of HM2 implantation to assess the impact of the learning curve on outcomes: the early-era group (from 2009 to 2014) (n=25), or the late-era group (from 2015 to 2017) (n=26). The cutoff point between years 2014 and 2015 was chosen arbitrarily as it coincided with reaching half of the total surgical case volume and thus allowed us to compare two groups with similar sample sizes.

### Surgical Technique for HM2 Implantation

The implantation technique of the HM2 remained constant throughout this study and was consistent with previous descriptions^[12]^. All patients underwent a median sternotomy. A preperitoneal pocket was created and the driveline was tunneled through the rectus muscle before heparin injection. Cardiopulmonary bypass (CPB) was used in all cases, but the aorta was clamped only if a concomitant intracardiac procedure was needed (n=12, 24%). The “core then sew” method was performed in all cases and 12 sutures of 2-0 Ethibond were placed in a pledgeted, interrupted, horizontal mattress fashion to attach the apical cuff. The inlet cannula was positioned within the left ventricle and the pump was placed in the preperitoneal pocket. The outflow graft was sewn in the ascending aorta in all cases. After deairing through the outflow graft, the patient was gradually separated from CPB, and the pump speed was progressively increased under echocardiographic guidance. Operative data are presented in Table 1.

**Table 1:** Postoperative in-hospital outcomes and complications following HeartMate II implantation by treatment era.

Variables (mean±SD or n [%])	Cohort n=51	Early n=25	Late n=26	*P*-value
In-hospital mortality	8 (16)	4 (16)	4 (15)	0.95
Length of ICU stay, days	15±15	14±13	18±17	0.40
Length of hospital stay, days	59±41	63±53	55±26	0.48
**Postoperative treatment requirements**				
Milrinone	41 (80)	16 (64)	25 (96)	0.16
Milrinone duration, days	4.5±3	4.0±4	4.9±3	0.50
Epinephrine	40 (78)	16 (64)	24 (92)	**0.04**
Epinephrine duration, days	5.1±6	2.6±1	7.5±8	0.05
Norepinephrine	43 (84)	17 (68)	26 (100)	**0.04**
Norepinephrine duration, days	7.7±12	4.1±6	10.9±15	0.13
NO	33 (65)	9 (36)	24 (92)	**0.01**
NO duration, days	3.2±2.7	2.5±3	3.7±3	0.39
Vasopressin	32 (63)	12 (48)	20 (77)	0.45
Sildenafil	25 (49)	5 (20)	20 (77)	**0.006**
**Bleeding complications**				
Tamponade	15 (29)	7 (28)	8 (31)	0.83
Major bleeding	20 (40)	13 (52)	7 (27)	0.07
Bleeding leading to death	4 (8)	3 (12)	1 (4)	0.64
Bleeding leading to reoperation	17 (33)	11 (44)	6 (23)	0.95
**Kidney complications**				
AKI	24 (48)	13 (52)	11 (42)	0.49
CVVH	11 (22)	6 (24)	5 (19)	0.56
Hemodialysis	5 (10)	3 (12)	2 (8)	0.61
Maximum creatinine	170±88	229±120	147±44	0.05
**Respiratory complications**				
Tracheostomy	4 (8)	3 (12)	1 (4)	0.28
Respiratory failure requiring MV	7 (14)	5 (20)	2 (8)	0.20
**Thrombotic complications**				
Arterial peripheral embolization	2 (4)	2 (8)	0 (0)	0.14
Stroke	5 (10)	4 (16)	1 (4)	0.15
**Infection complications**				
Sepsis requiring IV antibiotics	14 (27)	6 (24)	8 (31)	0.59
Sepsis leading to death	2 (4)	0 (0)	2 (8)	0.16
Pneumonia	11 (22)	3 (12)	8 (31)	0.10
Driveline infection	1 (2)	1 (4)	0 (0)	0.30
**Right heart failure**				
Inotropic support >7 days	19 (37)	10 (40)	9 (35)	0.69
Mechanical support	4 (8)	3 (12)	1 (4)	0.28

ACI=acute kidney injury; CVVH=continuous venovenous hemofiltration; ICU=intensive care unit; IV=intravenous; MV=mechanical ventilation; NO=nitric oxide

### Outcome Definitions

The primary endpoint was all-cause mortality. One-year and 4-year survival rates were examined. Early outcomes were defined as complications occurring during the immediate postoperative hospitalization or within 30 days of implantation. A composite endpoint was used to examine adverse outcomes at 90 days, including mortality, documented neurological events (*i.e.*, stroke or transient ischemic attack), reoperations for bleeding, need for a temporary right ventricular assist device (RVAD), and pump thrombosis. The 90-day timeframe was chosen because this is the period during which adverse outcomes after CF-LVAD implantation are more likely to occur, after which the risks decrease^[13]^.

### Statistical Analysis

Data are presented as mean±standard deviation (SD) or as median and range for normally and non-normally distributed continuous variables, and as frequencies with percentages for categorical variables.

To compare the two groups, univariate analysis was performed using chi-square tests for categorical variables, unpaired t-tests for continuous variables with equality of variances, and Mann-Whitney U tests for continuous variables without equality of variances. Actuarial survival curves were estimated using the Kaplan-Meier method and were compared between subgroups using the log-rank test.

A cumulative sum (CUSUM) control chart was used in order to assess trends in postoperative outcomes throughout this study^[14]^. This allows the graphical representation of the accumulated difference between the observed outcome and the target outcome. In this study, when the composite endpoint (*i.e.*, 90-day mortality, neurological event, reoperation for bleeding, need for a temporary RVAD, or pump thrombosis) was reached for a case, a sharp increase is observed in the graphic, whereas when a case does not reach the composite endpoint, this expected outcome is represented by a decrease in the curve.

Statistical analyzes were performed using GraphPad (Prism 6, GraphPad® software, San Diego, California) and SPSS (IBM Corp. Released 2019. IBM SPSS Statistics for Mac, Version 26.0. Armonk, NY: IBM Corp). All analyzes were conducted at a significance level of 0.05.

## RESULTS

### Patient Characteristics

Pre- and perioperative characteristics of patients in the two eras are presented in Table 2. In the entire cohort, 28 patients (55%) were implanted as BTT, with no difference between the two eras (early: n=15, 60% *vs.* late: n=13, 50%; *P*=0.47).

**Table 2:** Patient characteristics and perioperative data in the early (2009 to 2014) and late (2014 to 2017) eras of HeartMate II implantations.

Variables (mean±SD or n [%])	Cohort n=51	Early n=25	Late n=26	*P*-value
BTT	23 (45)	10 (40)	13 (50)	0.47
**Demographic data**				
Age, years	58±11	58±12	58±10	0.77
Men	41 (80)	19 (76)	22 (85)	0.43
BMI, kg/m^2^	25.8±4	25.9±4.7	25.6±3.6	0.40
**Medical history**				
Insulin-treated diabetes	4 (8)	4 (16)	0 (0)	**0.01**
Obesity	9 (17)	5 (20)	4 (15)	0.66
PVD	4 (8)	1 (4)	3 (11)	0.31
COPD	11 (21)	3 (12)	8 (31)	0.10
CKD	24 (47)	12 (48)	12 (46)	0.89
History of neoplasia	12 (22)	8 (32)	4 (15)	0.16
AF or flutter	26 (51)	13 (52)	13 (50)	0.89
Previous stroke	9 (18)	6 (24)	3 (12)	0.29
Modified Rankin Scale 0		5 (20)	3 (12)	
Modified Rankin Scale 1		1 (4)	0 (0)	
PH	21 (41)	11 (44)	10 (39)	0.68
Previous cardiac surgery	6 (12)	4 (16)	2 (8)	0.35
**Medications at home before admission**				
ACE inhibitor	26 (51)	13 (48)	13 (50)	0.88
ARBs	10 (19)	5 (20)	5 (19)	0.95
Beta-blocker	39 (76)	15 (60)	24 (92)	**0.007**
MRas	30 (61)	12 (48)	18 (69)	0.12
Warfarin	23 (46)	14 (56)	9 (35)	0.12
Loop diuretics	42 (84)	18 (72)	24 (92)	**0.05**
Dosage, mg/day	100±70	123±77	82±61	0.25
Current ICD	38 (74)	20 (80)	18 (70)	0.37
CRT	26 (51)	12 (48)	14 (54)	0.67
**Clinical state at admission**				
INTERMACS profiles 2 or 3	32 (63)	16 (64)	16 (62)	0.89
INTERMACS profiles 4, 5, or 6	13 (25)	6 (24)	7 (27)	0.81
Hospitalizations in the previous year	2.6±1.2	2.7±1.2	2.5±1.3	0.64
NYHA IV	11 (22)	5 (20)	6 (23)	0.49
Inotropes	37 (73)	20 (80)	17 (65)	0.39
ECMO support	5 (10)	3 (11)	2 (9)	0.60
**Primary diagnosis**				
Ischemic cardiomyopathy	18 (35)	10 (45)	8 (31)	0.76
Idiopathic cardiomyopathy	16 (31)	6 (27)	10 (39)	0.42
Familial cardiomyopathy	4 (8)	2 (9)	2 (7)	0.78
Toxic cardiomyopathy	4 (8)	2 (9)	2 (8)	0.82
Other	6 (12)	2 (7)	4 (17)	0.54
**Biological parameters**				
Serum creatinine, µmol/L	143±45	148±48	139±42	0.62
Total bilirubin, mg/dL	24.7±16	23±16	24±15	0.93
NT-proBNP, ng/L	9,028±6,939	8,777±6,300	9,265±7,598	0.77
**Echocardiography**				
LVEF, %	18.7±5.4	19.3±6	18.2±5	0.29
LVEDD, mm	70±9	74±8	67±10	0.26
**RV function**				
Normal function	13 (26)	7 (29)	6 (23)	0.52
Mild dysfunction	20 (40)	8 (33)	12 (46)	0.16
Moderate dysfunction	13 (26)	6 (25)	7 (27)	0.89
Severe dysfunction	4 (8)	3 (13)	1 (4)	0.25
Moderate or greater TR	11 (22)	5 (20)	6 (23)	0.51
Moderate or greater MR	17 (33)	8 (32)	9 (34)	0.97
Moderate or greater AR	2 (4)	0 (0)	2 (9)	0.14
**Right heart catheterization**				
PSAP, mmHg	51±12	52±13	50±11	0.38
mPAP, mmHg	34.3±8.2	33±8	35±8	0.54
CI, L/min/m^2^	2.1±0.7	2.3±0.7	2.0±0.7	0.53
PVR, Woods	3.1±1.5	2.3±1.7	2.9±1.3	0.10
RAP, mmHg	10.5±6.1	11.4±6	9.5±5	0.97
PCWP, mmHg	25±7	24±7	26±6	0.82
PAPi	3.6±2.2	3.6±2.3	3.5±2.0	0.15
RVSWI, g/m^2^/beat	13.5±5.3	14.9±5.8	12.4±4.8	0.16
**Perioperative data**				
CPB time, min	88±35	89±38	87±32	0.90
Aortic cross-clamp	10 (20)	2 (8)	8 (31)	**0.04**
Cross-clamp time, min (10 patients)	36±23	71±9	27±13	**0.007**
Concomitant procedure	22 (44)	8 (32)	14 (54)	0.11
CABG	6 (12)	3 (12)	3 (13)	0.89
Cryoablation	3 (6)	2 (7)	1 (4)	0.82
Aortic valve closure	2 (4)	0 (0)	2 (9)	0.31
Tricuspid valve repair	3 (6)	0 (0)	2 (9)	0.31
Other	9 (18)	3 (12)	6 (23)	0.30
VV ECMO for right heart failure	4 (8)	2 (7)	0 (0)	0.26

ACE=angiotensin-converting enzyme; AF=atrial fibrillation; AR=aortic regurgitation; ARBs=angiotensin-receptor blockers; BMI=body mass index; BTT=bridge to transplantation; CABG=coronary artery bypass grafting; CI=cardiac index; CKD=chronic kidney disease; COPD=chronic obstructive pulmonary disease; CPB=cardiopulmonary bypass; CRT=cardiac resynchronization therapy; ECMO=extracorporeal membrane oxygenation; ICD=implantable cardioverter-defibrillator; LVEDD=left ventricular end-diastolic diameter; LVEF=left ventricular ejection fraction; mPAP=mean pulmonary artery pressure; MR=mitral regurgitation; MRAs=mineralocorticoid receptor antagonists; NT-proBNP=N-terminal (NT)-pro hormone BNP; PAPi=pulmonary artery pulsatility index; PCWP=pulmonary capillary wedge pressure; PH=pulmonary hypertension; PSAP=pulmonary systolic artery pressure; PVD=peripheral vascular disease; PVR=pulmonary vascular resistance; RAP=right atrial pressure; RV=right ventricular; RVSWI=right ventricular stroke work index; TR=tricuspid regurgitation; VV ECMO=venovenous ECMO

Patients in the early era had a higher rate of diabetes and lower rates of beta-blockers and loop diuretics. Their rate of previous stroke was also double that of the late era, but the patients had comparable modified Rankin Scales in both groups. Most of the strokes were thought to be cardioembolic in nature and associated with a concomitant diagnosis of atrial fibrillation. Primary diagnosis, Interagency Registry for Mechanically Assisted Circulatory Support (INTERMACS) profiles, biological and echocardiographic parameters and right heart catheterization values were similar between the two eras. CPB time was similar between the two eras, whereas more patients required a concomitant cardiac procedure in the late era.

### Postoperative Complications

In-hospital outcomes and complications in the two eras are presented in Table 2. No difference was observed in terms of in-hospital mortality, length of ICU and hospital stay, and complications. In the late era, right ventricular dysfunction was treated more systematically, with 92% of patients using epinephrine, 100% norepinephrine, 92% nitric oxide, and 77% using sildenafil after HM2 implantation. However, the rate of right heart failure (defined as the need of inotropic support longer than 7 days or the need for mechanical circulatory support) was not different. During follow-up, 9 patients (18%) had pump thrombosis (early: n=4, 16% *vs.* late: n=5, 19%), and 3 patients underwent pump replacement in the late era. Twenty-four patients (47%) underwent HT, with no difference between the two eras (n=13, 52% *vs.* n=11, 42%; *P*=0.49).

The composite endpoint of 90-day mortality, reoperation for bleeding, need for temporary RVAD, neurological dysfunction, and pump thrombosis was significantly higher in the early-era group compared to the late-era group (n=17, 68% *vs.* n=9, 35%; *P*=0.02) (Table 3). However, none of the individual components were significantly different. The most important driver for this difference in the composite endpoint is likely reoperation for bleeding (n=11, 44% *vs.* n=6, 23%; *P*=0.11).

**Table 3:** Composite endpoint and outcomes at follow-up in the early (2009 to 2014) and late (2015 to 2017) groups of HeartMate II implantation.

Variables, n (%)	Cohort n=51	Early n=25	Late n=26	*P*-value
Composite endpoint (within 90 days)	26 (51)	17 (68)	9 (35)	**0.02**
90-day mortality	10 (20)	6 (24)	4 (15)	0.43
Reoperation for bleeding	17 (33)	11 (44)	6 (23)	0.11
Need of temporary right ventricular assist device	4 (8)	3 (12)	1 (4)	0.27
Neurological dysfunction	7 (14)	4 (16)	3 (11)	0.64
Pump thrombosis	2 (4)	1 (4)	1 (4)	0.98

RVAD=right ventricular assist device

### Survival After CF-LVAD Implantation

Despite a longer follow-up in the early era (57±41 months *vs.* 39±20 months, *P*=0.05), the duration of HM2 support was not different (524±70 days *vs.* 678±663 days, *P*=0.43).

**Fig. 1:**
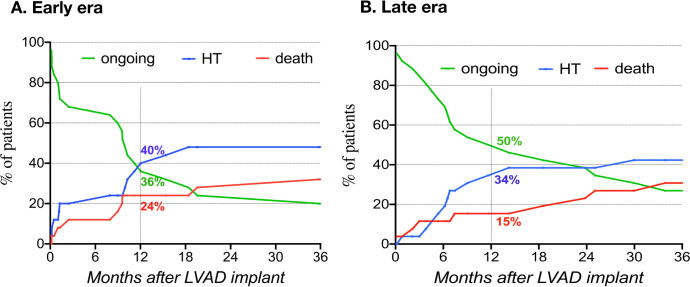
Competing outcomes after left ventricular device implantation in the early (A) and late (B) eras.

At one year, 18% of patients had died, 39% underwent a HT, and 43% were on CF-LVAD support. Competing outcomes curves in the early and late eras were not significantly different (Figure 1). During the study follow-up, 14 patients (56%) died in the early-era group and 8 patients (31%) died in the late-era group (*P*=0.06). Four patients (16%) died after HT in the early era. The main contributing causes of death were multiple organ failure, major bleeding, stroke and right heart failure and did not differ between the two groups (Table 4).

**Table 4:** Contributing causes of death in patients by treatment era.

Variables (mean±SD or n [%])	Cohort n=51	Early n=25	Late n=26	*P*-value
Overall death at the end of follow-up	22 (43)	14 (56)	8 (31)	
Multiple organ failure	11 (50)	7 (50)	4 (50)	1.0
Support withdrawal (palliative care)	11 (50)	4 (29)	7 (88)	0.008
Right heart failure	5 (23)	3 (21)	2 (25)	0.85
Major bleeding	6 (27)	4 (29)	2 (25)	0.86
Neurologic	8 (36)	3 (21)	5 (63)	0.05
Respiratory failure	5 (23)	4 (29)	1 (13)	0.39
Arrhythmia	2 (9)	2 (14)	0 (0)	0.26
Sepsis	2 (9)	0 (0)	2 (25)	0.05

**Fig. 2:**
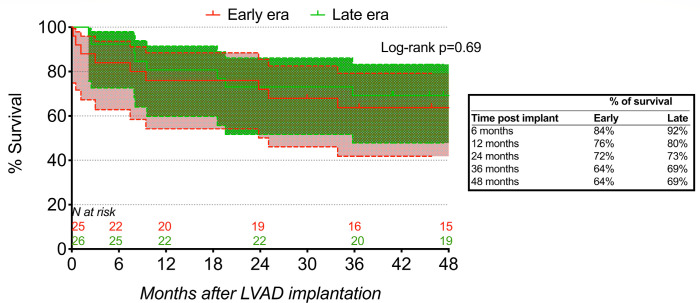
Kaplan-Meier survival curves after HeartMate II implantation in the early and late eras.

Survival by Kaplan-Meier analysis at 6 months, 1 year, and 4 years was 84%, 76% and 64% in the early era and 92%, 80%, and 69% in the late era (Figure 2). Although the 5-year postoperative follow-up has not yet been reached for 8 patients in the late-era group, 14 patients in the early-era group (n=14/25, 56%) and 17 patients in the late-era group (n=10/18, 56%) were alive after five years.

### Learning Curve for CF-LVAD Implantation

**Fig. 3:**
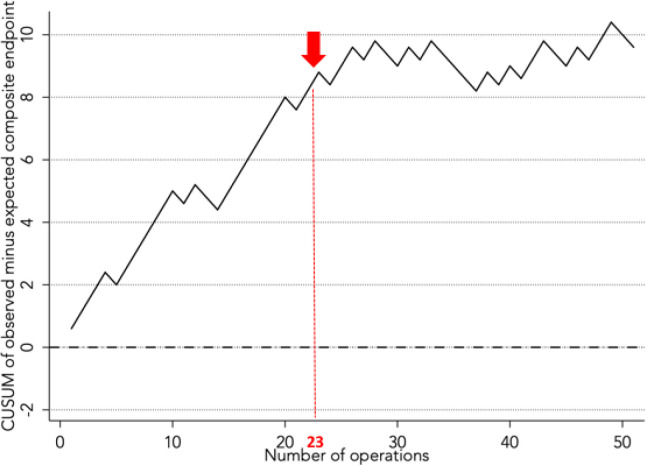
CUSUM analysis of outcomes between observed and expected rates of reaching the composite endpoint (i.e., 90-day mortality, neurological event, reoperation for bleeding, need for a temporary right ventricular assist device, or pump thrombosis).

A CUSUM analysis was conducted to analyze the incidence of the composite endpoint throughout this study (Figure 3). In the early stages of the study, there was a gradual increase in observed composite endpoint, but the curve levels out after reaching 23 operations. This suggests that 23 operations could be the threshold at our center after which outcomes are optimized.

## DISCUSSION

Although some publications have demonstrated a relationship between volume-center/expertise and survival following CF-LVAD implantation, the CUSUM analysis is an original way of illustrating this phenomenon in a homogeneous CF-LVAD population, consisting of only HM2 implantations in a low-volume center. Patients in the late era had a lower rate of comorbidities such as diabetes or previous stroke and were more likely to be treated with epinephrine, nitric oxide, norepinephrine, or sildenafil during the immediate postoperative period. A lower incidence of the composite endpoint was found in the late-era group compared to the early-era group, suggesting that results have improved over time at our institution. This was confirmed by a CUSUM analysis in which optimal results were obtained following 23 CF-LVAD implantations.

The association between the quality of outcomes and the cases volume has been shown in several cardiac surgery subspecialties. In an INTERMACS analysis, Cowger et al.^[4]^ found that center volume correlates with post-CF-LVAD survival, with worse outcomes in very-low-volume centers(<10 cases). Many other studies have previously demonstrated that a greater volume of patients correlates with decreased risk of adverse outcomes, with some suggesting that a center should have between 20 to 50 annual implantations per center^[1,4-7,9]^. This was reinforced by the inclusion of center volume in the HM2 Risk Score as a significant risk factor for 90-day mortality. Low-volume center in their case was defined as centers with <15 implantations during the trial. However, the notion that the learning curve could influence outcomes is important. With improvements in hospital processes, there can be reductions in rates of acute kidney injury and infection. Likewise, adherence to the PREVENT study recommendations regarding implantation technique, anticoagulation regimens, and pump speeds can reduce incidence of pump thrombosis^[15]^. Additionally, the concept of heart failure teams with specialized clinicians managing these patients improves outcomes in mechanical circulatory support, including in low-volume centers^[16]^.

Our center had an annual volume of approximately 4 implants per year in the early era and 6.5 implants in the late era. As a result, it is a very low-volume center with <10 implants per year. Despite this low annual procedural volume, our outcomes remain comparable to those reported in the INTERMACS database, with similar 1-year and 5-year survivals, 78% *versus* 84% (*P*=0.34) and 56% *versus* 46% (*P*=0.15) respectively^[17]^. In addition, we observed a drop in composite endpoint incidence between the two eras. This is consistent with the findings from Mussivand et al.^[18]^, who found that total center volume correlated with outcomes, while annualized frequency did not. Possible factors for the improvement in outcomes are patient selection and surgical technique. Between the two eras, there was a concurrent change in patient selection towards patients with less comorbidities and with a more optimized medical therapy which may have improved outcomes. These changes may be part of the institutional learning curve in the management of these patients. As for surgical technique, the HM2 was the sole CF-LVAD device used in our center during the study period, thereby allowing greater focus on mastering this device. In addition, reoperation for bleeding was the likely driver of the composite endpoint between the two eras. This may be related to one of two changes. First, the surgical team may have become more comfortable with medical management of certain bleeds, hence reducing the number of returns to the operating room. Second, we have implemented multiple measures over the years to decrease the rate of postoperative bleeding: achieving a more meticulous intraoperative hemostasis and a less aggressive anticoagulation strategy (*i.e.*, delaying intravenous heparin by up to 24 hours, not giving dipyridamole in addition to heparin). Otherwise, the surgical steps and operative technique have not changed between the two eras. This is similar to other cardiac surgery procedures which improve with the procedural learning curve, such as Ross procedures^[19]^, minimally invasive mitral valve surgery^[20]^, and frozen elephant trunk operations^[21]^.

In the secondary analysis, patients who had a CF-LVAD implant as DT were older and had a lower survival rate. This lower survival rate in patients with DT is well known in the literature and was highlighted in previous INTERMACS reports^[22]^. The 8^th^ annual INTERMACS report has shown that the survival rate in patients with a BTT and DT device strategy at implantation were 85% and 75%, respectively, at one year and 76% and 62%, respectively, at two years in the early era of the registry^[13]^. This discrepancy did not change much with time depending on the era of implantation^[22]^. Again, this compares to our own cohort which had an overall survival of 78% at one year.

With the constant renewal of medical technology, there is a gradual shift towards newer generation CF-LVAD devices such as the HeartMate 3, which presents better outcomes than the HM2^[23]^. The HM2 is therefore progressively replaced in favor of these better-performing devices in developed countries. This was the case in our center, which has stopped HM2 implantation in 2018. Results like those reported in this study remain important in the wider picture of international cardiac surgery. Indeed, there remains a significant unmet need for cardiac surgery in developing countries, in which costs, among other factors, become a limiting factor for access to healthcare^[24]^. As such, the improvement in outcomes with the HM2 based on the learning curve is encouraging given its future use primarily in low-volume centers around the world.

### Limitations of the Study

This study carries all the limitations of a retrospective single-center study with a small number of patients. There may therefore be under-reporting of certain outcomes such as driveline infection if these events occurred in another hospital center and were not reported in the patient files at our institution. The two eras have been arbitrarily separated in the middle of the study period according to number of implantations. The HM2 is a second-generation pump that is no longer used in most ventricular assist device centers. Outcomes after CF-LVAD implantation are highly related to many factors that might not be included in our study^[10]^. These include patient selection and referral, patient comorbidities, medical optimization before CF-LVAD implantation, surgical technique, immediate pre- and postoperative management, and the expertise of the team involved in the management of these patients. Our study did not adjust for all the changes that could have occurred during the study period and the difference in outcomes might be related to the difference in patient characteristics in addition to the learning curve effect. Most CF-LVAD implantations were performed by a single surgeon, and these results might be attributable to their personal learning curve.

## CONCLUSION

In our experience as a low-volume center, the learning curve influences postoperative complications, but not survival, in HM2 implantation with a threshold of 23 cases for optimized results, possibly through improvements in patient selection and surgical technique. Patients undergoing HM2 implantation for BTT also had better long-term outcomes than patients who underwent HM2 implantation for DT, likely due to less comorbidities in the former.
